# Personality preference influences medical student use of specific computer-aided instruction (CAI)

**DOI:** 10.1186/1472-6920-6-7

**Published:** 2006-02-01

**Authors:** John A McNulty, Baltazar Espiritu, Martha Halsey, Michelle Mendez

**Affiliations:** 1 Department of Cell Biology, Neurobiology and Anatomy, Loyola University Stritch School of Medicine, Maywood, IL 60153 USA; 2 Department of Medicine, Loyola University Stritch School of Medicine, Maywood, IL 60153 USA; 3 Learning Assistance Center, Loyola University Stritch School of Medicine, Maywood, IL 60153 USA; 4 Department of Preventive Medicine and Epidemiology, Loyola University Stritch School of Medicine, Maywood, IL 60153 USA

## Abstract

**Background:**

The objective of this study was to test the hypothesis that personality preference, which can be related to learning style, influences individual utilization of CAI applications developed specifically for the undergraduate medical curriculum.

**Methods:**

Personality preferences of students were obtained using the Myers-Briggs Type Indicator (MBTI) test. CAI utilization for individual students was collected from entry logs for two different web-based applications (a discussion forum and a tutorial) used in the basic science course on human anatomy. Individual login data were sorted by personality preference and the data statistically analyzed by 2-way mixed ANOVA and correlation.

**Results:**

There was a wide discrepancy in the level and pattern of student use of both CAI. Although individual use of both CAI was positively correlated irrespective of MBTI preference, students with a "**S**ensing" preference tended to use both CAI applications more than the "i**N**tuitives". Differences in the level of use of these CAI applications (i.e., higher use of discussion forum vs. a tutorial) were also found for the "**P**erceiving/**J**udging" dimension.

**Conclusion:**

We conclude that personality/learning preferences of individual students influence their use of CAI in the medical curriculum.

## Background

Computer-aided instruction (CAI) has become an increasingly important component of the medical curriculum due in large part to the development of Internet applications and the ease with which curricular content is distributed through networks [1-4]. The rapid increase in development of educational software and the more recent explosion in information databases available through the Internet have provided easy access to educational materials that have enhanced a student's abilities to learn either in small groups or individually with increased efficiency, better outcomes, and at reduced costs [5].

However, our data indicate that students are not uniformly making use of computer resources. Recent studies have revealed a wide disparity in utilization by individual students [6,7], which was attributed to differences in student attitudes toward computer technology [7]. Evidence in favor of this hypothesis was provided by a follow-up study showing that the degree to which individual medical students accessed the medical school network was related to their personality preferences as measured by the Myers-Briggs Type Indicator (MBTI) test [8].

The MBTI has been used extensively to measure the personality profiles of medical students [9-12] and describes eight preferences within four separate dimensions [13,14]. "**I**ntroversion" vs. "**E**xtroversion" is the dimension that describes a person's focus of attention and source of energy, whether from within or from the outside world. "I**N**tuition" vs. "**S**ensing" is the dimension that describes how an individual processes information either by focusing on the relationships between facts or the facts themselves. "**F**eeling" vs. "**T**hinking" is the dimension that describes whether decisions are made subjectively and personally or objectively and logically. The final dimension, "**P**erceiving" vs. "**J**udging", determines whether an individual's preference is to be spontaneous and flexible or decisive and orderly. The MBTI test is now generally accepted as a useful tool to help predict learning styles as well [15].

This study was designed to further test the hypothesis that personality preference is an important factor in the utilization of CAI in a medical curriculum. The specific objective of the study was to evaluate the extent to which personality preference influenced utilization of two different Web-based CAI applications developed for the M1 (first year) course in human anatomy. The two CAI applications differed in the level of user interactivity and the degree to which each application was directly applicable to learning objectives in the course.

## Methods

First year undergraduate medical students who were subjects in the present study took the MBTI test through the Office of Learning Assistance. The median age was 23 (21–31 range) and 48% of the students were female.

One of the CAI applications tested was the "LUMEN Forum", which allowed asynchronous communications among course faculty and students utilizing WebBoard conferencing server software (). The software included a variety of administrative tools for content management allowing delivery of multiple conferences that were individually tailored to address specific threaded discussions of general concepts and topics covered in the course. This application was highly interactive because the students contributed information whether in the form of text, or links to multimedia (images, videos, etc) as part of threaded discussions. The discussions were not always directly applicable to learning objectives in the course.

The second CAI application was "LUMEN Flash", a tutorial that provided a series of questions on a specific subject matter for review. This CAI was developed using ColdFusion (Allaire/Macromedia) and SQL (Structured Query Language) environments for delivering database driven applications through the Web. The applications created tables after each student access to record which "stack" of cards the student requested and which "cards" the student marked as correct or which cards/questions the student missed. This tracking allowed the student, upon returning to "LUMEN Flash" at a later time, to select only those cards s/he had previously not seen or had marked as being incorrect. The application included 7 general subject categories from which the students could select. The number of questions ("cards") in the categories ranged between 30 and 48 (mean = 39). The "LUMEN Flash" application was less interactive than the "LUMEN Forum" since students did not contribute to the program, but the scope of the categories were directly applicable to the learning objectives in the course.

Students were required to login to both CAI applications before use. Login data were stored for individual students in entry logs. The frequency with which students posted on the "LUMEN Forum" was determined directly from the postings by individual students in each conference. For the "LUMEN Flash" application, logins in which a student attempted to answer less than 10 questions in any session were excluded as incomplete sessions based on discussions with those students who explained that these sessions were usually terminated due to distractions or other time demands. All data were entered into Excel spreadsheets. Once the data for individual students were entered, the names of students were deleted from the database prior to further analyses in order to maintain the confidentiality of individual students. The study was exempted by the IRB.

Means and standard errors are reported for all of the grouped data. A 2-factor mixed ANOVA was used for data analyses. Pearson correlations (r) were also used to describe associations between continuous variables. The MBTI yields 16 separate personality groupings, but statistical analyses of these groupings were not conducted because of the very small sample sizes in many of the groups.

## Results

A total of 116 students were included in the analyses. The distribution of MBTI personality preferences (Table 1) showed that the majority of students exhibited preferences for "**E**xtroversion" (68%), "i**N**tuition" (55%), "**F**eeling" (66%), and "**J**udging" (58%).

There was a wide range in the frequency of logins among individual students ranging from 0–49 for the "LUMEN Forum" and 0–38 for the "LUMEN Flash". The overall frequencies of logins for each of the CAI applications were significantly different with the average access of the "LUMEN Forum" being higher (13.6 ± 1.0 sem) compared to the "LUMEN Flash" (9.5 ± 0.7 sem). Use of the "LUMEN Forum" was significantly higher regardless of the MBTI dimension (Table 1).

Three (3) students never logged into the "LUMEN Forum" compared to 17 students who never logged into the "LUMEN Flash". In spite of these differences, when the entire study group was considered, the frequency with which individual students logged into both CAI was positively correlated (p < 0.001) (i.e., the frequency of access to one CAI was positively associated with the frequency of access to the second CAI). Positive correlations were also found when the frequencies of logins to both CAI were analyzed according to MBTI preferences (Table 2). Failure to achieve significance in the "Thinking" preference was probably due to the relatively small N in this group.

When the data were sorted by personality preferences, 2-way ANOVA revealed an effect of MBTI preference for the "i**N**tuitive/**S**ensing" dimension. Students with "**S**ensing" preferences utilized the "LUMEN Forum" and the "LUMEN Flash" more frequently than those with "i**N**tuitive" preferences (Table 1). An effect of personality preference on student utilization of specific CAI was further indicated by interaction effects for the "Perceiving/Judging" dimension. In this case, "Judgers" tended to prefer the "LUMEN Forum" application to a much greater degree than the "LUMEN Flash" compared to "**P**erceivers" (Table 1).

The pattern of use of "LUMEN Flash" varied considerably among individual students, ranging from those who used the CAI to review all of the questions in relevant subject categories only once prior to each exam to those students who reviewed subject categories several times during the course. The pattern of use of the "LUMEN Forum" was also quite variable. As noted above, most students (97%) logged into the Forum, but the frequency of logins ranged from 1 to 49. Of those who logged in, relatively few (34%) contributed to the discussions and the frequency with which these individuals posted ranged from 1 to 28. When the data were sorted according to MBTI preferences, the strongest associations between frequency of logins and postings were found for the "**I**ntrovert", "**T**hinking" and "**P**erceiving" preferences (Table 3).

## Discussion

Three general observations are gleaned from the results of our study. First, there was a wide disparity in student use of CAI that was specifically developed to facilitate instruction of the subject matter. Second, there was a generally strong correlation in the level of individual use of respective CAI and this association was not dependent on MBTI personality/learning preference (with the possible exception of the "Thinking" preference). Third, the level of CAI use was influenced by MBTI personality/learning preferences.

To our knowledge, this is the first study that has examined effects of personality/learning preferences on utilization of specific CAI in the context of the medical curriculum. The finding that students with a "**S**ensing" preference tended to log in more frequently to both CAI is noteworthy because "**S**ensing" individuals characteristically focus on facts and conceptualizing through practical applications, which are traits consistent with the nature of both applications. The "LUMEN Flash" was mostly factual and the "LUMEN Forum" frequently provided practical applications of important anatomical concepts. Further effects of MBTI personality/learning preferences on the level of use of the respective CAI were found when comparisons were made between CAI (e.g., "**J**udgers" tended to use the "LUMEN Forum" more frequently than "LUMEN Flash").

Our observation that the "**S**ensing/i**N**tuitive" dimension influenced use of CAI is consistent with a report by Ahn [16] who found that students with "**S**ensing" preferences were more satisfied with CAI used in distance education. Friend and Cole [17] also reported that "**S**ensing/**T**hinking" individuals responded best to CAI compared to "i**N**tuitive/**F**eeling" students. In another study, the "**T**hinking/**F**eeling" preference was the most significant dimension affecting use of CAI and the development of declarative knowledge [18]. Smith et al. [19] reported that "i**N**tuitive/**T**hinking" types of teachers were more likely to use technology than the "**S**ensing/**F**eeling" types. The "i**N**tuitive/**T**hinking" types also learned significantly better using CAI designed to teach language [20]. Students with "**F**eeling" preferences had poorer attitudes toward technology and made more mistakes using CAI than those with a "**T**hinking" preference [21]. In an earlier study, we also reported that students who accessed the medical education network more frequently tended to have a "**T**hinking" preference [8]. The results of the present study cannot be compared directly with our previous study because that study did not examine utilization of any specific CAI; it only measured general use of the computer. Much of the variability reported in the literature probably relates to differences in the paradigms used in the evaluations.

With the advent of Web-based instruction, which promotes non-linear interactions with most CAI, research has begun to focus on how individual learner differences influence the use of these instructional paradigms (cf. [22]). The wide disparity in frequency of CAI use among individual students was found in an earlier study in which computer use by individual medical students was quantified [8]. Coates and Humphrey [6] also reported that students in an economics class exhibited similar variability in use of on-line practice quizzes and discussion boards. In this case, 18% never attempted any of the practice quizzes and only 25% of students read more than a quarter of all postings in the discussion forum. Although our study suggests that innate personality/learning preferences may explain differences in the level of use of CAI by students in the medical curriculum, it does not exclude many other factors (e.g., background, prior experiences. cognitive styles, etc), which presumably also have an influence.

Our study did not specifically address important questions related to summative evaluations, but the findings are relevant assuming that the degree of utilization of specific CAI applications is correlated with outcomes on in-course examinations as demonstrated in a previous study [23]. An important advantage of the experimental paradigm for future studies is that measurements of effectiveness of CAI as related to performance in class (knowledge retention) are fully integrated into the day-to-day real-life curriculum of the students.

## Conclusion

We conclude that personality/learning preferences affect the level of use of CAI specifically developed for use in the medical curriculum. More broadly, our results are linked to the overall theory that instruction is most effective when it "fits" with the individual student's needs. Because a student's approach to learning predicts academic achievement, it is of obvious importance to tailor computer applications to the individual student's intellectual and psychological profile using CAI that is founded in basic principles of learning and instructional design. This concept was emphasized by Pocius [24], who stated that, "Understanding the effects of personality variables on computer use can be used to improve the quality of human-computer interaction. The awareness of which personality traits introduce individual differences in human-computer interactions can illuminate ways in which a particular human-computer interaction task can be altered to accommodate different users."

## Competing interests

The author(s) declare that they have no competing interests.

## Authors' contributions

JM originated the idea for the research, oversaw the collection and analysis of data, and wrote the manuscript. BE assisted with development of the CAI and revised the manuscript. MH assisted with collection and interpretation of the MBTI data, and revising the paper. MM assisted with analysis of the data and revising the manuscript. All authors have read and approved the final manuscript.

## Pre-publication history

The pre-publication history for this paper can be accessed here:



## Figures and Tables

**Table 1:** Frequency of logins for each of the four MBTI dimensions. Means (± sem) for utilization of the specific CAI applications tested in this study. MBTI preferences exhibiting higher values for each CAI are bolded to highlight trends.

	**Forum Logins**	**Flash Logins**	**ANOVA Main Effects**		
			
			Applications	MBTI Preference	Interactions
**I**ntrovert n = 37	13.2 (± 2.0)	9.4 (± 1.3)	p < 0.001	p = 0.831	p=0.883
Extrovert n = 79	**13.8 (± 1.2)**	**9.6 (± 1.0)**			
i**N**tuition n = 64	11.1 (± 1.1)	8.2 (± 0.9)	p < 0.0005	p = 0.006	p = 0.240
**S**ensing n = 52	**16.6 (± 1.8)**	**11.2 (± 1.3)**			
**F**eeling n = 77	13.5 (± 1.2)	**9.7 (± 1.0)**	p < 0.0005	p = 0.885	p = 0.768
**T**hinking n = 39	**13.6 (± 2.1)**	9.1 (± 1.3)			
**P**erceiving n = 49	11.5 (± 1.4)	**10.2 (± 1.3)**	p < 0.001	p = 0.443	p = 0.032
**J**udging n = 67	**15.1 (± 1.5)**	9.1 (± 1.0)			

**Table 2:** Correlation of individual logins to "Forum" and "Flash" CAI.Coefficients (r) and p values for correlations between the frequency of logins to the "LUMEN Forum" vs. the "LUMEN Flash" when individual students were sorted by personality preference.

**Group**	**r**	**p**
**I**ntrovert	0.35	0.03
**E**xtrovert	0.39	0.001
i**N**tuitive	0.27	0.03
**S**ensory	0.42	0.002
**F**eeling	0.45	0.001
**T**hinking	0.25	0.13
Perceiving	0.40	0.004
**J**udging	0.39	0.001

**Table 3:** Correlation between logins and postings to the "LUMEN Forum".Coefficients (r) and p values for correlations between the frequency of logins vs. postings to the "LUMEN Forum" when individual students were sorted by personality preference.

	**r**	**p**
**I**ntrovert	.509	0.001
**E**xtrovert	.267	0.017
i**N**tuition	.389	0.001
**S**ensing	.221	0.108
**F**eeling	.182	0.109
**T**hinking	.543	0.001
**P**erceiving	.409	0.003
**J**udging	.227	0.067
